# Bioactivity of Sodium Free Fluoride Containing Glasses and Glass-Ceramics

**DOI:** 10.3390/ma7085470

**Published:** 2014-07-25

**Authors:** Xiaojing Chen, Xiaohui Chen, Delia S. Brauer, Rory M. Wilson, Robert G. Hill, Natalia Karpukhina

**Affiliations:** 1 Dental Physical Science, Institute of Dentistry, Barts and the London School of Medicine and Dentistry, Queen Mary University of London, Mile End Road, London E1 4NS, UK; E-Mails: xiaojing.chen@qmul.ac.uk (Xiaoj.C.); xiaohui.chen@qmul.ac.uk (Xiaoh.C.); r.hill@qmul.ac.uk (R.G.H.); 2 Otto-Schott-Institut, Friedrich-Schiller-Universität, Fraunhoferstr. 6, Jena 07743, Germany; E-Mail: delia.brauer@uni-jena.de; 3 School of Engineering and Materials Sciences, Queen Mary University of London, Mile End Road, London E1 4NS, UK; E-Mail: r.m.wilson@qmul.ac.uk

**Keywords:** sodium-/alkali-free bioactive glass, bioactive glass-ceramics, fluorapatite, fluoride

## Abstract

The bioactivity of a series of fluoride-containing sodium-free calcium and strontium phosphosilicate glasses has been tested *in vitro*. Glasses with high fluoride content were partially crystallised to apatite and other fluoride-containing phases. The bioactivity study was carried out in Tris and SBF buffers, and apatite formation was monitored by XRD, FTIR and solid state NMR. Ion release in solutions has been measured using ICP-OES and fluoride-ion selective electrode. The results show that glasses with low amounts of fluoride that were initially amorphous degraded rapidly in Tris buffer and formed apatite as early as 3 h after immersion. The apatite was identified as fluorapatite by ^19^F MAS-NMR after 6 h of immersion. Glass degradation and apatite formation was significantly slower in SBF solution compared to Tris. On immersion of the partially crystallised glasses, the fraction of apatite increased at 3 h compared to the amount of apatite prior to the treatment. Thus, partial crystallisation of the glasses has not affected bioactivity significantly. Fast dissolution of the amorphous phase was also indicated. There was no difference in kinetics between Tris and SBF studies when the glass was partially crystallised to apatite before immersion. Two different mechanisms of apatite formation for amorphous or partially crystallised glasses are discussed.

## 1. Introduction

The bioactivity of glasses and ceramics is often investigated on immersion of the solid in a buffer solution simulating physiological fluids. Formation of an apatite-like layer on the surface of the solid as a result of the reaction with fluid is then evaluated. This laboratory procedure has a number of drawbacks and cannot be used directly for predicting the bioactivity of the same solid *in vivo* [1,2]. However, testing apatite formation in buffer solution is a useful inexpensive model for comparative evaluation of the bioactive glasses and bioceramics of different formulations in order to preselect compositions with potentially the most promising biological activity. 

The earliest immersion time when apatite can be identified is typically used as a measure of bioactivity. However, the rate and ability of bioceramics to form apatite is dependent on the type of physiological buffer used even when the same formulation of bioceramics or glass is tested. In particular, concentrations of calcium and phosphate available in solution as well as the presence of other species affect the rate of apatite formation. The composition of the simulated body fluid (SBF) solution is close to the saturation level of apatite [2]. Therefore, potentially any surface exposed to SBF can aid precipitation of apatite irrespective of whether it is bioactive or not. On the other hand, only calcium- and phosphate-rich bioceramics and glasses can be tested for bioactivity in Tris buffer solution, owing to apatite precipitation following the initial release of these components from the solid into solution. 

Alkali- or specifically sodium-containing bioactive glasses generate a high alkaline pH that can cause a cytotoxic cells response [3]. Although plenty of alkali-free bioceramics [4,5] based on calcium-rich crystalline phases have been studied including phosphates [6], carbonates [7] and silicates [8,9], there are only few reports on alkali-free bioactive glasses [10,11,12,13,14,15,16]. The majority of the available sodium-free bioactive glasses are of sol-gel origin [17,18,19,20,21,22], and the nominal composition of these glasses cannot be directly compared with melt-derived glasses owing to the presence of substantial amounts of, if not water, then hydroxyl groups in the glass. In a report where exclusively sodium-free melt-derived glasses were studied, the bioactivity of the glasses was clearly inhibited owing to the presence of significant amounts of MgO in the composition [12].

Typically, the rate of apatite formation is lower for ceramics than for amorphous glasses, as a crystalline solid has a lower dissolution rate and thus would be expected to exhibit lower bioactivity than an amorphous glass of a bioactive-type composition. For instance, sintered apatite bioceramics dissolves slower than apatite resulting from the degradation of bioactive glass [23]. Sintered glass exhibits a bioactivity lower than the one before sintering [24]. Moreover, partial crystallisation often occurs during sintering, for instance with 45S5, which will further decrease the reactivity of the glass [25,26,27].

Recent success in fluoride-containing bioactive glasses showed that there is a concentration limit for fluoride to be added to the glass before spontaneous crystallisation occurs [10,11,28,29,30]. This is owing to the known effect of fluoride destabilising glass against crystallisation [31]. It is also known that the type of the fluoride-containing crystalline phase is dependent on the composition of the glass. For example, in low phosphate content bioactive glasses, calcium fluoride crystallises out [32]. An increase in phosphate content caused formation of apatite in the glasses [33]. 

In the present study, the design of glasses is typical for bioactive compositions [34,35] and it is such that the silicate component is present mostly as chains [32,36,37], while fluoride is coordinated by calcium [38] and phosphate is present as orthophosphate [32,36]. Partial crystallisation of an apatite phase should not involve the silicate chain domains [32,33,38,39], and these will still remain amorphous, which means the silicate phase would still continue to dissolve rapidly. In addition, apatite crystals pre-formed in the system can minimise the nucleation energy via providing their surface for further precipitation and growth of the apatite crystals. Although apatite-containing systems have been known before, none of the bioactivity studies considered this in combination with a reactive glass phase. We propose that the bioactivity of these glasses will not be significantly affected by partial crystallisation of the glasses.

Thus, the aim of this paper is to investigate the bioactivity of sodium-free amorphous or partially crystallised glasses and compare the bioactivity of the two.

## 2. Results and Discussion

### 2.1. Apatite Formation

#### 2.1.1. X-ray Diffraction (XRD) Results

**Figure 1:**
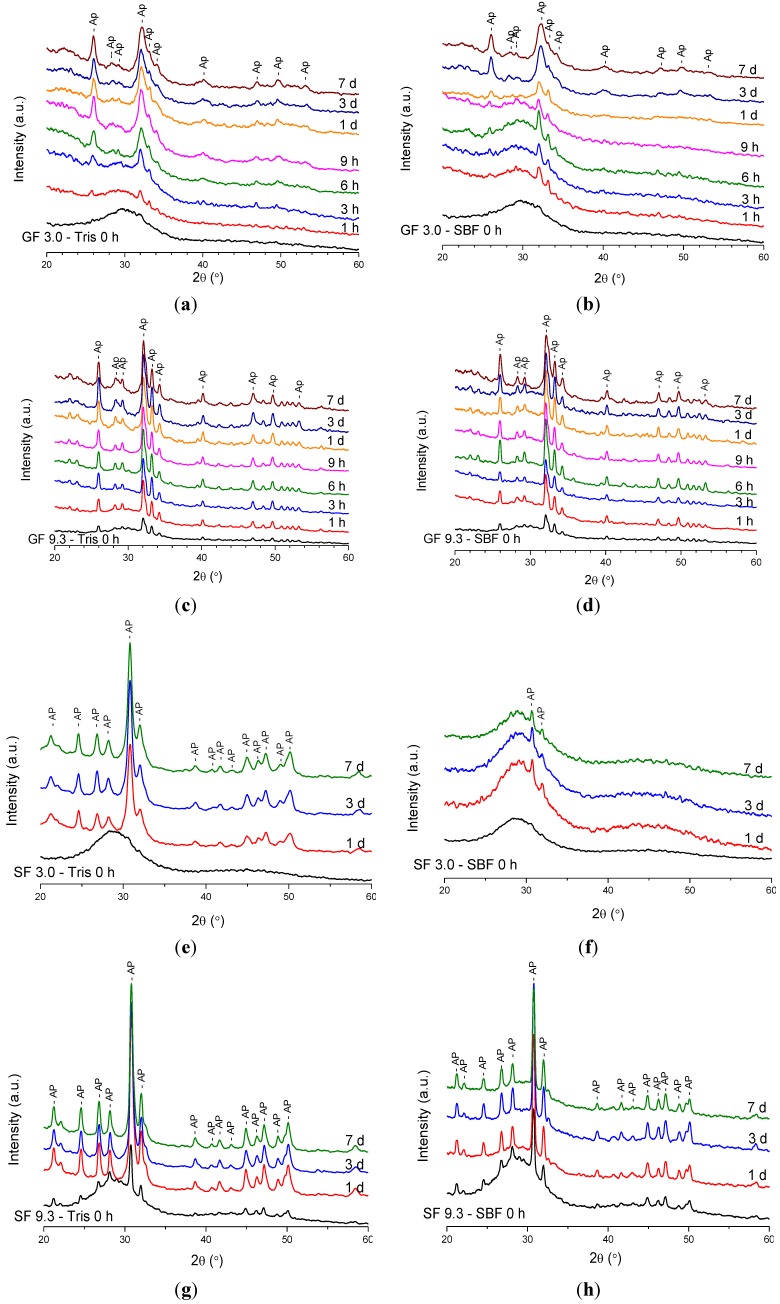
XRD patterns of glasses after immersion in buffer solutions: (**a**) GF 3.0 in Tris; (**b**) GF 3.0 in SBF; (**c**) GF 9.3 in Tris; (**d**) GF 9.3 in SBF; (**e**) SF 3.0 in Tris; (**f**) SF 3.0 in SBF; (**g**) SF 9.3 in Tris; (**h**) SF 9.3 in SBF. Label Ap in CaF_2_ series is fluorapatite Ca_5_(PO_4_)_3_F (00-034-0011), and strontium fluorapatite Sr_5_(PO_4_)_3_F in SrF_2_ series (00-050-1744).

XRD results of four compositions after immersion in Tris and SBF solutions are presented in Figure 1a–h. The compositions with 3 mol% CaF_2_ and SrF_2_ were amorphous as quenched, whereas compositions with 9.3 mol% CaF_2_ and SrF_2_ were partially crystallised upon quenching.

In Figure 1a,b, clear characteristic apatite peaks developed at 25.9° and 31.8° 2θ after short immersion times. The intensities of these diffraction lines increased with immersion time. However, it is seen that the intensities grew more rapidly in Tris buffer compared to SBF. The presence of significant amounts of apatite is revealed in the XRD patterns after immersion in Tris between 3 and 9 h, whereas a similar intensity of the apatite diffraction lines is seen after immersion in SBF for 3 days only. Immersions for durations longer than 9 h in Tris and 3 days in SBF did not reflect significant changes on the XRD patterns. Similar results were obtained for compositions with 0, 4.5, and 6 mol% CaF_2_ (not shown).

Glasses with higher CaF_2_ content that had partially crystallised to mainly fluorapatite (FAP) also exhibited bioactivity. As seen from Figure 1c,d, the intensity of apatite peaks increased after immersion compared to the non-immersed glass. However, there was no significant difference in the intensity of the apatite diffraction lines obtained after immersion in Tris and SBF for the same time period. It is clearly seen that the apatite diffraction lines are significantly broader for the compositions with low CaF_2_ content (Figure 1a,b) than for the compositions with high CaF_2_ content (Figure 1c,d) owing to the presence of crystalline FAP in the untreated glasses with high CaF_2_ content.

Figure 1e,h show the XRD patterns of SrF_2_-containing glasses after immersion, and the results are similar to those seen in the CaF_2_ series. The diffraction peaks at 30.5° and 31.7° 2θ, which correspond to strontium phosphate fluoride (Sr_10_(PO_4_)_6_F_2_), developed after immersion of the composition with 3 mol% SrF_2_ in Tris and SBF (Figure 1e,f). The intensity of the apatite diffraction lines after immersion in Tris is much stronger than after immersion in SBF. However, the difference in intensity after immersion in Tris or SBF is not significant when the SrF_2_-containing glass contains crystalline fractions of apatite as observed for the composition with 9.3 mol% SrF_2_ (Figure 1g,h).

Thus, for both the CaF_2_ and SrF_2_ series, the fraction of apatite detected from the XRD patterns grew faster during immersion in Tris than in SBF solution when the immersed glass powders were XRD amorphous before immersion. The higher initial intensity of the apatite lines for the partially crystallised glasses indicates that the apatite crystals present in the untreated glass served as nuclei for the apatite phase formed during immersion in buffer solution, and newly formed apatite crystals precipitated on the surface of the pre-existing crystalline phase.

#### 2.1.2. Fourier Transform Infrared (FTIR) Spectroscopy Results

**Figure 2:**
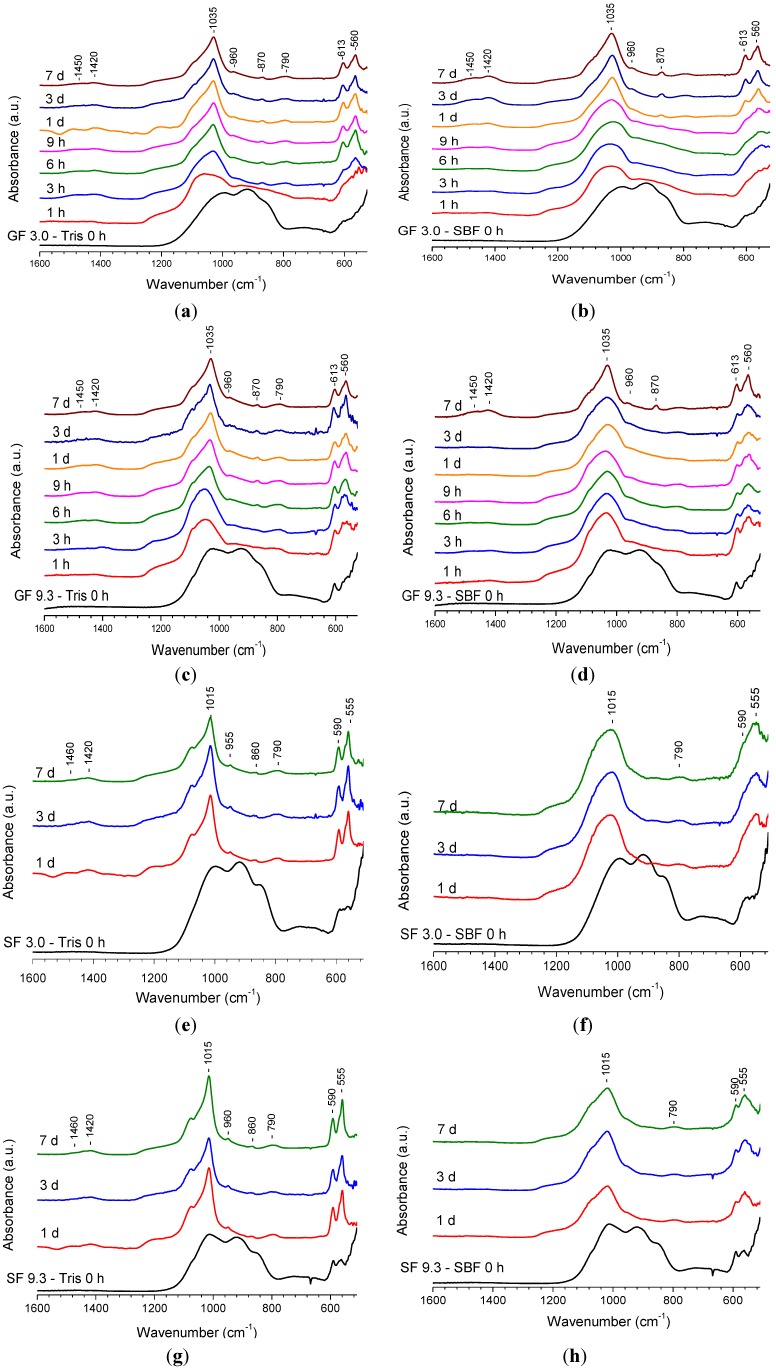
FTIR spectra of glasses after immersion in buffer solutions: (**a**) GF 3.0 in Tris; (**b**) GF 3.0 in SBF; (**c**) GF 9.3 in Tris; (**d**) GF 9.3 in SBF; (**e**) SF 3.0 in Tris; (**f**) SF 3.0 in SBF; (**g**) SF 9.3 in Tris; (**h**) SF 9.3 in SBF.

FTIR spectroscopy was used to monitor glass degradation and apatite formation upon immersion of glasses in buffer solution, and Figure 2a–h presents the FTIR results for the same four compositions shown in Figure 1. Each bottom spectrum in Figure 2 corresponds to the non-immersed glass powder mainly showing three strongly overlapped bands at about 1000, 920 and 870 cm^−1^, which are known for bioactive type silicate glass and are assigned to the Si-O vibration modes, *i.e.*, Si-O-Si stretch, non-bridging oxygen Si-O^−^ stretch and bend, respectively [10]. This assignment is consistent with the IR spectra computed by using accurate DFT calculations recently reported for phosphosilicate bioactive glasses [40,41]. The spectra of the samples collected after immersion reveal clear changes compared to the non-immersed glasses.

The non-bridging oxygen Si-O^−^ band at 920 cm^−1^ decreased in intensity immediately upon immersion and disappeared at longer immersion times. All the spectra clearly show sharpening of the band at about 1035 cm^−1^ which is associated with both Si-O-Si and crystalline orthophosphate. The latter additionally accounts for the shoulder appearing at about 1100 cm^−1^ [10,11]. However, it is seen that the sharpening occurs more rapidly during immersion in Tris rather than SBF. Very clear changes can be seen in the region 500–600 cm^−1^. The typical crystalline orthophosphate or apatite split bands at 560 cm^−1^ and 613 cm^−1^ are present for GF 3.0 glass at 3 h immersion in Tris (Figure 2a) and can be distinguished at 9 h of immersion in SBF (Figure 2b). The spectra indicating the presence of crystalline apatite have another band at about 960 cm^−1^ corresponding to the P-O stretch in the orthophosphate tetrahedron. The spectra for the samples collected after immersion contain bands at 1450, 1420 and 870 cm^−1^, which are associated with the presence of carbonate substitution in the apatite. The band at 790 cm^−1^ appearing in the spectra after immersion is assigned to Si-O-Si between the two adjacent silicate tetrahedra [11,42]. This forms as a result of condensation reactions on glass degradation and essentially presents a silica-gel. Figure 2a shows that starting from 6 h of immersion the spectra are very similar to each other. No changes can be seen in the spectra at 24 h of immersion in SBF (Figure 2b).

The evolution of bands in the FTIR spectra for the GF 9.3 composition after immersion in Tris and SBF (Figure 2c,d) is similar to the one observed for glass GF 3.0. The spectrum of the original glass reveals signs of the presence of crystalline FAP in addition to the typical bands of Si-O vibration. On immersion, the spectra clearly reveal changes owing to glass dissolution. In addition, it is seen that the sharpening of the bands occurs at earlier time points after immersion in Tris than in SBF. Similar results were obtained for other compositions with CaF_2_ contents higher than 9.3 mol% (not shown).

The FTIR spectra of SF 3.0 and SF 9.3 (Figure 2e–h) reveal similar results for the SrF_2_-containing series. Upon immersion, non-bridging oxygen band disappeared, and a crystalline orthophosphate band develops. The position of the split bands characteristic for crystalline orthophosphate is slightly different from the CaF_2_ series. These developed at 590 and 555 cm^−1^ which may be explained by the presence of strontium cations [39]. The FTIR spectra of SF 9.3 are similar to those of other compositions with SrF_2_ contents higher than 3.0 mol%. The differences in the rate of sharpening of the FTIR bands in Tris compared to SBF immersion is clearly seen in this series, too.

On comparison with the calcium series, it is seen that the spectrum of SF 3.0 immersed in Tris for 1 day (Figure 2e) displays sharper features than the spectrum of GF 3.0 at the same immersion time (Figure 2a), which indicates that the strontium-containing glass may dissolve and form apatite slightly faster than the calcium-containing one. FTIR spectroscopy characterises both amorphous and crystalline species present in solids unlike XRD, which reveals changes in the crystalline domains of a solid only. The presented FTIR results indicate that glass dissolution may occur faster in Tris buffer solution than in SBF for amorphous or partially crystallised bioactive silicate glasses.

#### 2.1.3. Magic Angle Spinning-Nuclear Magnetic Resonance (MAS-NMR) Spectroscopy Results

**Figure 3:**
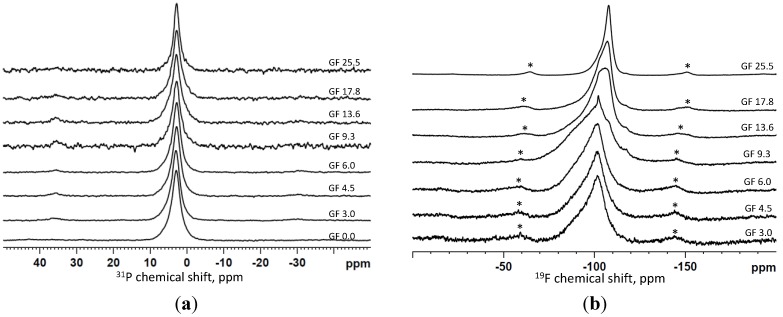
^31^P (**a**) and ^19^F (**b**) MAS-NMR spectra of the samples collected after immersion in Tris buffer solution for 6 h.

Figure 3 displays the ^31^P and ^19^F MAS-NMR spectra of the calcium fluoride-containing series immersed for 6 h in Tris buffer; the spectra are plotted for increasing fluoride content from bottom to top. The ^31^P MAS-NMR spectra in Figure 3a are very similar for the entire series and reveal a relatively sharp peak with the centre between 2.8 and 3.0 ppm, assigned to crystalline apatite [43]. Some of the spectra show an asymmetry of the signal indicating presence of multiple phosphorus species that can include the residual glass signal as well. The latter is expected to be around 3 ppm, though is broader than the signal assigned to apatite.

The ^19^F MAS-NMR spectra shown in Figure 3b revealed multiple overlapping signals including the crystalline species of a fluoride-substituted apatite with a signal at about −103 ppm and calcium fluoride at about −108 ppm characterised by a sharp peak [11]. No crystalline calcium fluoride appears to be presence in the spectra of the compositions with low fluoride content (GF 3.0, GF 4.5 and GF 6.0); these glass compositions showed only formation of fluorapatite during immersion. The asymmetry of the signal is explained by the presence of fluoride species in the residual glass; the spectra of the initial glasses are characterised by a broad signal at about −95 ppm [39]. Starting from composition GF 9.3 the sharp feature at about −108 ppm corresponding to CaF_2_ can be distinguished in the spectra (Figure 3b), increasing in intensity at higher fluoride contents in the glass. Finally, in composition GF 25.5, most of the fluoride is present as crystalline calcium fluoride at 6 h immersion in Tris, although a crystalline fluorapatite in addition to calcium fluoride and fluorine in the glass phase was detected in the original glass before immersion.

The MAS-NMR results in Figure 3 indicate that 6 h immersion of the glass powders in Tris buffer produced a fluoride-substituted apatite as the major solid phase in most of the compositions. Although this result is consistent with those obtained from XRD and FTIR, the results from ^19^F NMR in Figure 3b provide evidence that the apatite formed is fluoride-substituted. The ^31^P MAS-NMR spectra of this glass series at 9 h of immersion were similar to the spectra in Figure 3a (6 h), although the line width decreased slightly at 9 h of immersion compared to 6 h. However, in the glass with the highest fluoride content most of the fluoride was detected as crystalline CaF_2_ with only a small fraction of fluorapatite present. In addition, for the composition GF 25.5, the spectra of 6 and 9 h of immersion are practically identical. The latter is consistent with the XRD results, where the patterns were practically identical.

The ^31^P NMR spectra of the partially crystallised glasses that contained an apatite phase before immersions (GF 9.3, GF 13.6, GF 17.8) were deconvolved [44], and the results show an increase in the apatite fraction at 9 h of immersion compared to the immersion for 6 h and the fraction of apatite in the untreated glass. A fraction of 20%–30% of the original phosphorus in GF 9.3, GF 13.6, GF 17.8 glasses was crystallised to an apatite; this increased up to 30%–40% at 6 h of immersion, which further increased to 40%–50% at 9 h of immersion in Tris. This indicates that the crystalline apatite formed in the glass spontaneously during quenching can serve as a seeding phase for the apatite precipitating during immersion in physiological solutions. This may also affect the morphology of the apatite formed on precipitation; the bigger apatite crystals of a more regular shape that formed spontaneously during glass quenching can further grow during immersion in buffer solution, whereas small nanometre-sized apatite crystals were formed when an amorphous bioactive glass powder was immersed in buffer solution [45]. 

### 2.2. Dissolution Study

#### 2.2.1. pH Measurements Results

**Figure 4:**
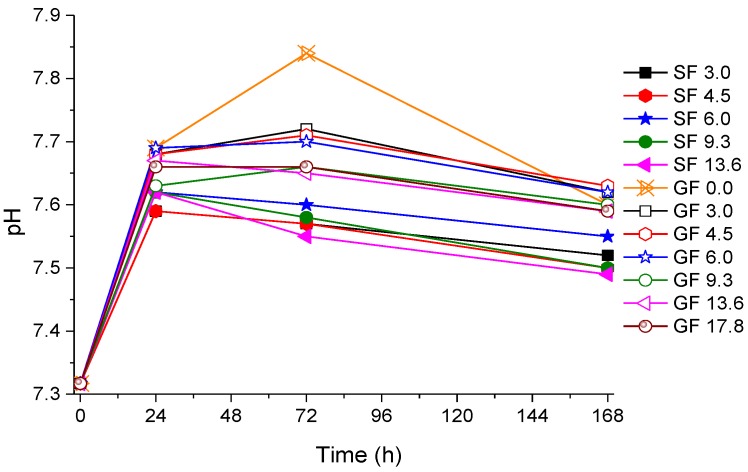
pH measured at the end of the immersion time in Tris buffer. Open symbols are for the CaF_2_ series (GF) and closed symbols are for the SrF_2_ series (SF) with the same nominal fluoride content.

Figure 4 shows pH changes in Tris buffer solution during immersion of glass powders measured at the end of the immersion time. The general trend is similar for all compositions: pH increased on initial immersion, which is explained by substitution of protons from the solution for the cationic components of the glass, which increases the alkalinity of the solution. However, as the apatite phase starts precipitating, pH does not increase any longer and may even slightly decrease. It is seen that the highest increase in pH at the initial stage was observed for the fluoride-free glass GF 0.0. This is consistent with the effect of fluoride incorporated into a bioactive glass to reduce an increase in pH on initial dissolution which was previously seen and discussed [11,46]. However, overall, it is seen that the strontium-containing glasses gave a slightly lower pH during dissolution (mostly between 7.5 and 7.6) than the calcium-containing ones (mostly between 7.6 and 7.7).

#### 2.2.2. Ion Release Results

**Figure 5:**
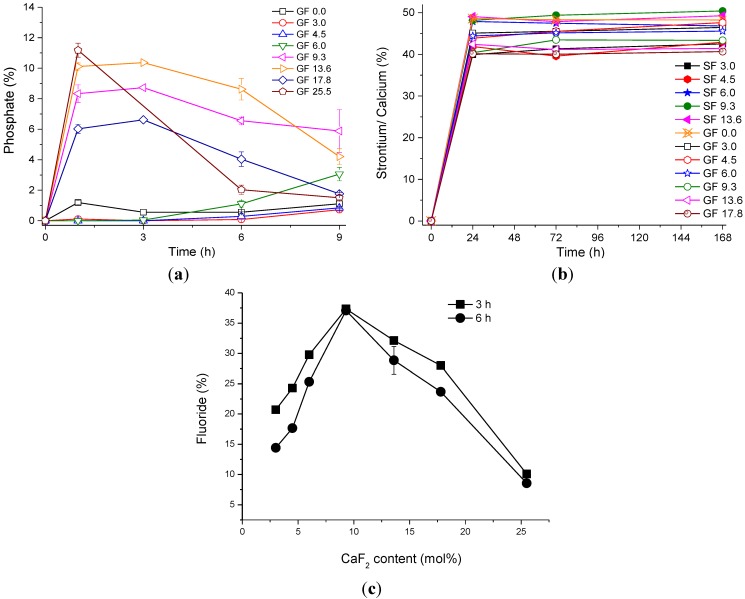
(**a**) The percentage of phosphate presented as concentration of elemental phosphorus in Tris for CaF_2_-containing glasses; (**b**) The percentage of cations (Sr^2+^ or Ca^2+^) concentration in Tris; (**c**) The percentage of fluoride concentration measured at 3 and 6 h in Tris for CaF_2_-containing glasses.

Figure 5a presents the relative phosphate concentration in Tris solution at 9 hours’ immersion of glass powders of the calcium fluoride series, presented as a percentage of the phosphate in the nominal glass composition. It is seen that two different trends are observed depending on whether the original glass was amorphous or contained a crystalline phase. Amorphous glasses with low fluoride content show only presence of very small percentages (less than 2%) of phosphate in solution. By contrast, the partially crystallised glasses containing high amounts of fluoride show higher concentrations (up to 11% of phosphate) at early time points (1 h), followed by a decrease in phosphate concentration, which is consistent with the precipitation of an apatite phase during which phosphate is consumed. The higher the fluoride content in the glass, the higher the phosphate concentration at 1 h. However, the subsequent drop in the release was most pronounced for the GF 25.5 glass composition.

The concentrations of calcium and strontium from the glass powders upon immersion up to 1 week are presented in Figure 5b. It is seen that at 24 h of immersion 40%–50% of calcium and strontium are present in solution, and this concentration does not change significantly at the later time points. There seems to be no trend with fluoride content.

The percentage of fluoride present in Tris buffer is shown in Figure 5c for 3 and 6 h of immersion. It is seen that the highest concentration (over 35% of the nominal fluoride content in the glass) was observed for composition GF 9.3. However, the lowest concentration of fluoride (about 10%) is seen for the glass with the highest fluoride content, GF 25.5, although most of the fluoride in the original composition was in the crystalline phase as seen from its ^19^F MAS-NMR spectrum. There was only a small difference between fluoride concentrations at 3 and 6 h of immersion, which is at the level of accuracy of the measurements. Thus, it is seen that fluoride concentrations increase with increase in fluoride content in the glass as long as fluoride remains in the amorphous glass. Once a fluoride-containing phase starts crystallising from the glass the fluoride concentrations decrease. This suggests that the amorphous fluoride-containing glass phase is the source of the fluoride release rather than the crystalline phase.

### 2.3. Final Discussion

This study presents bioactivity data for two fluoride-containing series of glasses that were either amorphous or partially crystallised. Those glasses which had partially crystallised can be considered as fluoride-containing glass-ceramics. All the glasses and glass-ceramics showed fast glass degradation and apatite formation upon immersion in buffer solutions. However, the results indicate that there is a clear difference in mechanism of bioactivity depending on whether the glass was amorphous or contained a fluoride-containing crystalline phase.

Based on FTIR, XRD and ^31^P MAS-NMR data, the initially amorphous glasses containing relatively low amounts of fluoride (below 9% in Ca-containing and below 4.5% in Sr-containing series) degraded rapidly upon immersion in Tris buffer and formed apatite within 3 h. Results from ^19^F MAS-NMR revealed that the apatite, which formed within 6 h in Tris, was fluorapatite. No crystalline CaF_2_ phase was detected after immersion for glasses with low fluoride contents.

For dissolution studies in Tris buffer, the glass was the only source of phosphate for apatite formation. This proves that a high amount of phosphate present as orthophosphate in the bioactive glass is clearly beneficial for early apatite formation. The ionic concentration in solution confirm rapid glass degradation in Tris buffer. However, the results for phosphate were surprising, as concentrations observed for the initially amorphous glasses were very low, despite apatite having formed at very early immersion time points. This suggests that either phosphate was released at even earlier time points than the earliest investigated here (1 h), or apatite might form via a different mechanism than via dissolution-precipitation.

The kinetics of glass degradation and apatite formation was slower in SBF compared to Tris buffer for the initially amorphous glasses, as seen particularly from comparison of FTIR spectra. Apatite formation was noted in SBF by 24 h compared to 6 h in Tris. This delay can be explained by the higher ionic strength of the SBF solution, which alters the kinetics of glass degradation via inhibiting the dissolution of ionic species. The presence of Mg^2+^ in SBF is known to suppress apatite formation [47] and can additionally contribute to a delayed formation of apatite [48].

Fluorapatite was not the only crystalline phase found in the partially crystallised glasses. Strong presence of CaF_2_ dominated in compositions with the highest fluoride contents starting from composition GF 13.6 with fluorapatite being present as a minor phase. However, the results presented above show that the fraction of crystalline apatite had grown in these bioceramics after immersion. The intensity of the signal assigned to apatite increased from XRD and FTIR. In addition, ^31^P MAS-NMR suggested an increase of the apatite fraction after immersion in Tris.

The decrease in phosphate concentrations from 1 h in solution confirms a consumption of phosphate for apatite formation. The phosphate concentrations for the partially crystallised glasses was significantly higher than for the amorphous glasses, despite it being assumed that phosphate is released from the amorphous phase of the glass-ceramics rather than the crystalline. Despite this distinct difference, growth of the apatite fraction of the glass-ceramics is estimated to have occurred by 3–6 h, which is a similar time frame as for the amorphous glasses with low amounts of fluoride.

Presence of apatite crystals in the untreated materials, even in minor amounts compared to the amorphous part, clearly affected the bioactivity of the glass-ceramics during immersion. The observed increase in the apatite fraction with time indicates that the existing apatite crystals served as a seeding phase for further apatite crystal growth. As the amorphous phase of the glass-ceramics rapidly degraded with time in buffer solution, the additional apatite had precipitated from the solution via a dissolution-precipitation mechanism. Presence of a seeding phase reduces the energy required for formation of apatite nuclei, which is typically the limiting factor of apatite crystallisation. Comparison of apatite formation for (partially crystalline) glass-ceramics immersed in Tris and SBF showed that there was no significant difference in the kinetics between the two solutions unlike for the amorphous glasses. This is owing to elimination of the rate limiting stage of apatite nucleation for the partially crystallised glass-ceramics.

There was no significant difference between the Ca- and Sr-containing series, except for a higher tendency of the Sr-containing glasses to crystallise.

## 3. Experimental Section

### 3.1. Glass Preparation

A melt-quench method was employed to synthesise sodium-free, fluoride-containing bioactive glasses. The glasses were designed by introducing different amounts of CaF_2_/SrF_2_ into a SiO_2_-P_2_O_5_-CaO/SrO glass system (Table 1). In order to preserve the predominantly Q^2^ glass structure, calcium fluoride was added to the glasses with the ratios between other components kept constant.

**Table 1:** Glass compositions in mol%.

Glass code	SiO_2_	CaO/SrO	P_2_O_5_	CaF_2_/SrF_2_
GF/SF 0.0	38.1	55.5	6.3	0.0
GF/SF 3.0	37.0	53.9	6.1	3.0
GF/SF 4.5	36.4	53.0	6.0	4.5
GF/SF 6.0	35.9	52.2	5.9	6.0
GF/SF 9.3	34.6	50.4	5.7	9.3
GF/SF 13.6	32.9	48.0	5.5	13.6
GF/SF 17.8	31.4	45.7	5.2	17.8
GF/SF 25.5	28.4	41.4	4.7	25.5

A 200 g batch of analytical grade silica (Prince Minerals Ltd., Stoke-on-Trent, UK), calcium carbonate, calcium fluoride and phosphorus pentoxide (Sigma-Aldrich, Gillingham, UK) were weighed out, mixed and transferred into a 300 mL platinum/rhodium crucible, melted at 1550 °C (glasses with 0 and 3 mol% CaF_2_/SrF_2_) and 1500 °C (all other glasses) for 1 h in an electrical furnace (EHF 17/3, Lenton, Hope Valley, UK). The melted glasses were quenched into cold deionised water to suppress crystallisation. The as-quenched granular glass frits were dried overnight and ground into powder by using a Gyro mill (Glen Creston, London, UK) for two sets of 7 min. A mesh analytical sieve (Endecotts Ltd., London, UK) with a size of 45 µm was applied to obtain fine powder.

### 3.2. Bioactivity Testing and Ion Release Measurements

Glass and glass-ceramics bioactivity was tested in Tris buffer solution and SBF. SBF was prepared following the recipe described by Kokubo and Takadama [2] for the corrected SBF (c-SBF). The preparation of Tris buffer solution was according to that described by Mneimne *et al.* [10]. The specimens were prepared in duplicate; 75 mg of glass powder was dispersed in 50 mL of buffer solution and transferred to a shaking incubator (KS 4000i Control, IKA, Staufen, Germany) at 37 ± 1 °C at an agitation rate of 60 rpm for 1, 3, 6, 9, 24, 72 and 168 h.

pH was measured using an Oakton^®^ pH meter with 35811-71 pH electrode at the end of each immersion period. The solutions were filtered using the filter papers with pore size of 5–13 μm and precipitates were collected. The filtered solution was kept at 4 °C until further analysis.

The filtered solutions were acidified with 69% nitric acid to quantify ions concentrations using an inductively coupled plasma-optical emission spectroscopy (ICP-OES; Varian Vista-PRO, Yarnton, UK). The solutions were diluted by a factor 1:20 for measurements of the calcium and silicon content and no dilution was used for measurements of phosphate ion content. Calibrations were performed using diluted multi-element stock solution. Fluoride content in solution was quantified using a fluoride ion selective electrode (Orion 9609BN, 710A meter, South Burlington, VT, USA) and NaF stock solution was used for preparation of the fluoride-containing calibration solutions.

### 3.3. Characterisation of Apatite Formation after Immersion

The precipitates collected after the immersion were dried and analyzed by X-ray Diffraction (XRD), Fourier Transform Infrared Spectroscopy (FTIR) and solid state Nuclear Magnetic Resonance (NMR).

An X’Pert Pro X-ray diffractometer (PANalytical, Eindhoven, The Netherlands) with a copper (Ni-filtered Cu-Kα) X-ray source was employed to characterise the glasses, glass-ceramics and the samples collected after immersion in buffer solutions. The patterns of powder samples were recorded between 5 and 70° 2θ at a step size of 0.0334°. Calibration was carried out using NIST standard reference material 660a (lanthanum hexaboride). XRD data were analyzed using X’Pert HighScore Plus (v2.0, PANalytical, Almelo, The Netherlands) in conjunction with the ICDD database.

The samples collected after immersion were assessed using Fourier Transform Infrared Spectroscopy (Spectrum GX, Perkin-Elmer, Cambridge, UK). The data were recorded from 500 to 1600 cm^−1^ of wavenumber.

Powders of the CaF_2_-containing glasses after soaking in buffer solutions were investigated using solid state NMR on a 600 MHz (14.1T) Bruker NMR spectrometer (Bruker AV 600 NMR, Conventry, UK). ^31^P MAS-NMR was run at the 242.9 MHz resonance frequency using a standard single resonance Bruker probe in a 4 mm rotor at spinning conditions of 8 and 10 kHz. Sixteen scans were run with a recycle delay of 60 s for each sample. The chemical shift was referenced using the primary reference, 85% H_3_PO_4_. ^19^F MAS-NMR measurements were run at the 564.7 MHz resonance frequency using a standard double resonance Bruker probe with low fluorine background for a 2.5 mm rotor spinning at a speed of 18 kHz and 21 kHz. Typically, 32 or 64 scans were acquired with eight preliminary dummy scans and 30 s recycling delay. The chemical shift was referenced using the signal from 1M NaF solution scaled to −120 ppm relative to the CF_3_Cl primary standard. The free *dmfit* software [44] was used for deconvolution of the NMR spectra.

## 4. Conclusions

Amorphous glasses with low amounts of fluoride (below 9 mol% CaF_2_) degraded rapidly in Tris buffer solution and formed apatite within 3 h, which by 6 h could be identified as fluorapatite by ^19^F MAS-NMR. Glass degradation and apatite formation were significantly slower in SBF compared to Tris buffer.

Partially crystallised glasses (containing crystalline phases such as fluorapatite or calcium/strontium fluoride) showed degradation of the amorphous phase and an increase in fraction of apatite by 3–6 h in Tris and SBF buffers, with no difference in kinetics observed for the two solutions. The increase in apatite fraction is thought to occur by precipitation of apatite onto the surface of apatite crystals already present within the glass-ceramics. High phosphate concentrations at 1 h followed by a decrease were observed for the glass-ceramics, which is in contrast to the amorphous glasses. It is proposed that two different mechanisms of apatite formation occur, depending on presence or absence of apatite crystals in the original glass.
